# The intentions, needs, advantages and barriers: a survey of twenty-nine countries participating in the “Belt and Road Initiative” health cooperation

**DOI:** 10.1186/s41256-019-0109-z

**Published:** 2019-06-28

**Authors:** Yao Wang, Xiaohui Liang, Yuchen Liu, Yuanan Lu, Xiaojun Liu, Zongfu Mao

**Affiliations:** 1 Global Health Institute/School of Health Sciences, Wuhan University, Wuhan, 430061 China; 2 High School of Nanjing Foreign Language School, Nanjing, 210008 China; 3 Department of Public Health Science, University of Hawaii at Manoa, Honolulu, HI 96822 USA

**Keywords:** Belt and road initiative, Health cooperation, Needs, Advantages, Barriers

## Abstract

**Background:**

China launched the Belt and Road Initiative (BRI) aiming to develop a mutually-beneficial, sustainable trade model with partner countries. The processes of international health exchanges are key parts of how this model can be sustained into the future. This paper aims to contribute to broader understanding by exploring various intentions, needs, advantages and barriers of BRI countries and then define ways China can achieve better health cooperation.

**Methods:**

In this study, a survey questionnaire was developed to investigate the perspectives of 60 international experts from 29 BRI countries. The survey was distributed and collected during the “Belt and Road High-level Meeting for Health Cooperation”, held in Beijing in August 2017. The data were entered into EpiData 3.0 by two team members respectively and analyzed with SPSS22.0.

**Results:**

Altogether 58 valid questionnaires were obtained. More than 93% of participants showed their willingness to be part of BRI health cooperation. Frist, three key areas were identified, which are broadly defined as BRI country “needs”: (1) “Develop health industries”; (2) “Prevent and control infectious diseases”; and (3) “Provide health services”. Second, three “advantages” of participating in BRI Health Cooperation were found: (1) “The establishment of a long-term partnership”; (2) “Quality investment and financing system”; and (3) “The convenience of cooperation”. Third, two main cross-cultural “barriers” for cooperation were encountered: (1) “Cultural differences”; and (2) “Lack of communication platforms”.

**Conclusion:**

In this study, we have identified priority fields that China can collaborate with BRI countries and show its leadership. It is suggested that successful health cooperation between China and other BRI countries should focus on maximizing present health-industry partnerships, investment and financing, as well as overcoming cultural and communication barriers.

## Introduction

The Belt and Road (B&R) is the abbreviation of the Silk Road Economic Belt and the twenty-first Century Maritime Silk Road. In 2013, Chinese President Xi first put forward the Belt and Road Initiative (BRI), which aimed to develop cooperative partnerships with countries along the Belt and Road, and jointly build a community of interests, destiny and responsibility. At present, BRI has been supported by lots of countries and have expanded beyond the B&R boundaries to the world. One of the most important parts of BRI is health cooperation, which can be called BRI Health Cooperation and carries China’s commitment to BRI countries. BRI Health Cooperation is best illustrated by China’s “Three-Year Plan for the Implementation of the ‘Belt and Road Initiative’ Health Exchange and Cooperation (2015-2017)” (hereinafter referred to as the “Implementation Plan”). This plan, now shifting from a period of infancy to a more mature international presence, has already implemented 41 major projects and activities, with positive commercial and reputation outcomes [1, 2]. Beyond these initial steps of international health cooperation via the BRI framework, though, what is clear is the significant role China will take in directing future international health cooperation. It can be seen that the projects and activities China have already implemented with other BRI countries represent a foreshadowing of a profound change to global health governance.

However, these initial successes, and the continued expansion of BRI and participating country numbers [3], belie the various barriers that BRI Health Cooperation faces. Much of the current cooperation plans, projects and activities continue in a phase of infancy. China’s strategy of beginning health cooperation programs with important fulcrum countries that have strong political foundations and a base of cooperation [4] has inevitably led to decentralized health cooperation. Other challenges range from insufficient forms of cooperation to weak continuity of cooperation content [5]. At the same time, broader health cooperation issues have come to light, including the indistinct relationship of supply and demand, slow timeline of cooperation decisions, and weak cooperation initiatives. So, just as China’s role in shaping the direction of future global health governance is clear, China will need to better identify and understand the intentions, needs, advantages and barriers of partnership countries to achieve more efficient and sustainable processes of health cooperation.

Scholarly explorations of the BRI have mainly focused on analyzing the intention of BRI Health Cooperation. Explorations taken from a primarily Chinese perspective mostly focus on summarizing and comparing certain areas of health cooperation, notably health systems and policies, traditional medicine, infectious disease prevention and control, ability improvement and personnel training, and health development assistance [6, 7]. As such, there are gaps in scholarly research. These gaps are to do with the ways cooperation among countries along Belt and Road (B&R) is developing, including an understanding of their health industry needs. This study is designed to begin filling these gaps. It is intended as a step toward a deeper appreciation of the key factors shaping BRI Health Cooperation: intentions, needs, advantages and barriers. Results from the study can be taken as baseline information for decision makers and participants in health cooperation.

## Methods

### Context and study sample

In August 18–19, 2017, the “Belt and Road High-level Meeting for Health cooperation: towards a Health Silk Road” was organized by the National Health Commission (NHC) of the People’s Republic of China (P.R.C.) in Beijing. Participants were composed of government officials, representatives of international organizations such as the United Nations and the World Health Organization, as well as representatives of relevant government departments, scientific research institutions, non-governmental organizations, think tanks, and enterprises. Altogether 60 international experts participated in the study with the assistance of the International Exchange and Cooperation Center, NHC, P.R.C.

### Survey questionnaire

The survey has been approved by the Medical Ethics Committee of Wuhan University School of Medicine. The “‘Belt and Road’ National Health Cooperation Intention Survey” questionnaire was adapted from the “China-Africa Health Cooperation Intention and Needs Questionnaire” [8]. It was developed by Wuhan University Global Health Institute and went through several rounds of expert consultations. The questionnaire includes general information, such as country, unit, position, nature of the unit, and 10 items, including the cooperating country/province, cooperation area, cooperation intention and demand, and health cooperation advantages and barriers. It was adapted and developed according to “Belt and Road High-level Meeting for Health Cooperation: towards a Health Silk Road” conference participant characteristics.

### Data collection and processing

Before the survey is launched, investigators were trained about the study. A pre-test trail was also conducted by two-person groups. During the investigation, a “one-to-one” questionnaire survey method was taken up. It was distributed, answered, and collected upon completion. Once all the completed questionnaires were collected, they will be summarized, coded, and screened by an investigator. The criteria for questionnaire selection was based on the completion rate ≥ 80%, with missing values replaced by a median option. If, in the case of two or more participants of the same position level in one unit or institution both/all filled out the questionnaires, selection priority was given to those who had more experience with international communication and cooperation. At last, all the survey results were entered twice by two investigators, simultaneously. Computer logic error detection was used to ensure an accurate completion of the final database. A total of 60 questionnaires were distributed and 58 of them were considered valid, making the effective rate of the questionnaire reach 96.7%. As a result, the 58 questionnaires were collected and analyzed for our study purpose.

### Statistical analysis

The data was statistically analyzed using SPSS 22.0. The qualitative data is represented by frequency, and rate or composition ratio for statistical description.

## Results

The 58 participants were from 29 countries around the world: Africa (11 nations), Asia (9 nations), Europe (3 nations), North America (5 nations) and South America (1 nation). Among the 58 participants, 24 were from Asia (41.4%), 3 from Europe and 1 from South America. Considering the relatively low participation from Europe, North America and South America (that is, a total of 15 people, accounting for 25.9% of the total number of survey), these participants are referred to as a collective titled, “Western countries”.

The participants were more often associated with “Government departments” and “Medical institutions”, and they accounted for 67.2% of the total number. In comparison, “Universities and research institutes”, “Enterprises”, and “International organizations” accounting for only 32.7% (Table 1).

**Table 1:** Unit nature of the participants

Unit nature	N (%)
Government departments	22 (37.9)
Medical institutions	17 (29.3)
Universities and research institutes	10 (17.3)
Enterprises	5 (8.6)
International organizations	4 (6.9)
Total	58 (100.0)

### Willingness to BRI health cooperation

More than 90% of participants expressed interest in BRI Health Cooperation. In comparison, only 3.4% of participants, or two participants, said they would not be a part of BRI Health Cooperation (Table 2), specifically a scholar from the United States and a Centers for Disease Control (CDC) official from Sudan. Two participants from Georgia, a nurse and an international staffer, indicated that they were not sure whether their countries had any intention to participate.

**Table 2:** willingness of participation in BRI Health Cooperation

Options	N (%)
Yes	54 (93.2)
No	2 (3.4)
Not clear	2 (3.4)
Total	58 (100.0)

### The needs for BRI health cooperation

One of the key results of the questionnaire survey, “needs”, shows participants views on where and what BRI Health Cooperation should focus its attention on. A large number of participants believed the most important areas for cooperation were “Health industry” (48.3%), “Health Services” (44.8%), and “Prevention and Control of infectious diseases” (43.1%). The demand for “Traditional medicine” cooperation was found to be the lowest “need”. Notably, participants also suggested a “need” to establish a sustainable food system, a subject included in the “Other” categorization (Table 3).

**Table 3:** Contents of participation in BRI Health Cooperation

Contents	N (%)
Health industry	28 (48.3)
Health Services	26 (44.8)
Prevention and control of infectious disease	25 (43.1)
International development assistance	16 (27.6)
Personnel training / medical research	14 (24.1)
Health emergency / emergency medical assistance	11 (19.0)
Health care management	8 (13.8)
Traditional medicine	2 (3.4)
Others	1 (1.7)

### Regional needs

**Fig. 1:**
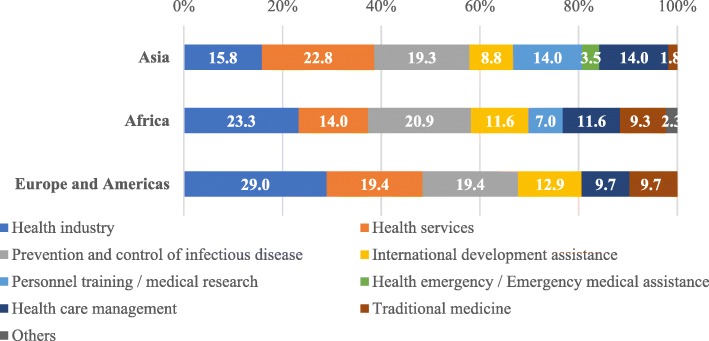
Areas of interest to cooperate with China in BRHC perceived by respondents from the countries of Asia (*n* = 24), Africa (*n* = 19), Europe and Americas (*n* = 15)

The survey also registered the “needs” associated with each specific region. For example, according to the survey, the greatest health cooperation “needs” in Asia are “Health services” (22.8%), “Prevention and control of infectious disease” (19.3%), and “Health industry” (15.8%). “Traditional medicine” (1.8%) was identified as being a non-priority “need”. For African and Western countries, the greatest “need” was “Health industry”. The second and third “needs” for these areas were “Prevention and control of infectious diseases” (20.9% and 19.4%, respectively), and “Health services” (14.0% and 19.4%). Neither area expressed a “need” to cooperate on “Health Emergency/Emergency medical assistance” (Fig. 1).

#### Advantages of BRI health cooperation

According to the participants, the three key advantages of health cooperation were (1) establish a “Long-term partnership” with China, (2) “A good investment and financing system” and (3) take advantage of the “Convenience of cooperation”. Conversely, issues such as “Preferential policies” and “Geographical advantages” were considered relatively disadvantageous, both 5.2% (Table 4).

**Table 4:** Advantages of participating in BRI Health Cooperation

Advantages	N (%)
Long-term partnership	34 (58.6)
A good investment and financing system	24 (41.4)
Convenience of cooperation	18 (31.0)
Medical services and product quality and reputation	15 (25.9)
The influence of Chinese traditional medicine	9 (15.5)
People’s acceptance to China	7 (12.1)
Preferential policies	3 (5.2)
Geographical advantages	3 (5.2)

### Regional advantages

**Fig. 2:**
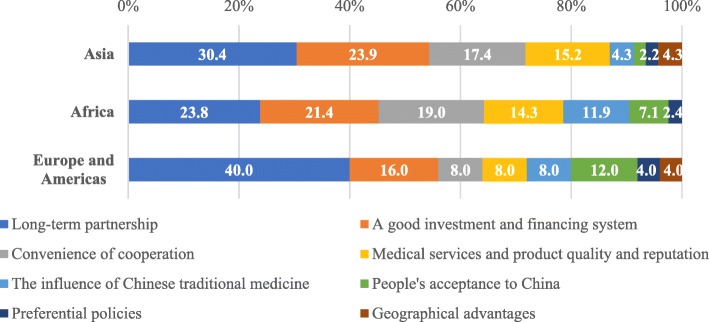
Advantages perceived by respondents from the countries of Asia (n = 24), Africa (n = 19), Europe and Americs (n = 15) on their countries’ participation in BRHC with China or the reasons for choosing China as the partner

The most important regional advantages were “Long-term partnerships” and “Good investment and financing systems”. Western countries considered “Long-term partnerships” to be more significant (40.0%). They also considered “Public acceptance to China” (12.0%) to be of greater advantage than Africa (7.1%) and Asia (2.2%). Each region identified “Preferential policies” and “Geographical advantages” as being a relatively low “advantage” (Fig. 2).

#### Barriers for BRI health cooperation

The major BRI Health Cooperation barriers, as noted by the survey respondents, were considered to be “Cultural differences” (34.5%) and “Lack of communication platforms” (32.8%). Conversely, the respondents considered “Legal challenge” (6.9%) and “Stability of policy environment” (6.9%) to be minor barriers. Another minor barrier, one not initially listed on the survey, was “Language barrier” (see “Other”: 5.2%), which the correspondents held that they were cognizant of during entire Sino-foreign cooperation projects (Table 5).

**Table 5:** Distribution of barriers in BRI Health Cooperation

Barriers	N (%)
Cultural difference	20 (34.5)
Lack of communication platform	19 (32.8)
Lack of e-trade platform	11 (19.0)
Lack of funds	10 (17.2)
Drug quality and certification issues	10 (17.2)
Complex application procedure	8 (13.8)
Lack of government support	7 (12.1)
The stability of the policy environment	4 (6.9)
Legal challenge	4 (6.9)
Others	3 (5.2)

### Regional barriers

**Fig. 3:**
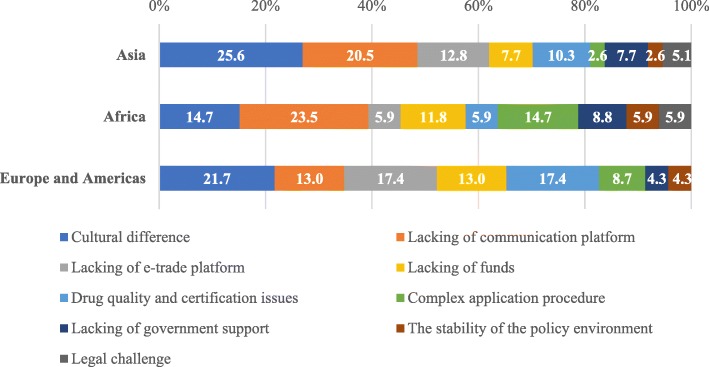
Barriers perceived by respondents from Asia (n = 24), Africa (n = 19), Europe and the Americas (n = 15) on their countries ‘participation in BRHC

In terms of regional specific responses, the Asian participants identified “Cultural difference” (25.6%), “Lack of communication platform” (20.5%), and “Lack of e-trade platform” (12.8%) as the major barriers of BRI Health Cooperation. For the Western country participants, the major barriers were similarly “Cultural difference” (21.7%) and “Lack of e-trade platform” (17.4%), as well as “Drug quality and certification issues” (17.4%). For the African participants, the greatest barriers were “Lack of communication platform” (23.5%), “Cultural difference” (14.7%), and “Complex application procedure” (14.7%) (Fig. 3).

## Discussion

### Broad prospects of BRI health cooperation

Since 1963, China has started to dispatch China Medical Team (CMT) on two-year rotations to provide clinical care in low-resource settings around the world, most of them in Africa. The program continues today in an expanded form: at any given time, more than 1000 staff are deployed abroad at an estimated cost of US$60 million annually [9]. This status reflects the critical shift in China’s role over the last decade from being an aid recipient to a non-conventional donor in global development aid; which is to reflect back on the fact that China is now playing an increasingly crucial role in global health development [10, 11].

In this context, our results highlight an overwhelming preference for cooperation (over 93%), which is notable on a number of levels, not least for the stark contrast it provides when viewed against recent global events and trends: as in, an economic recession in Europe, a rising trend toward anti-globalization, war and chaos in West Asia, and an economic downturn in Africa [12]. As such, the preference denotes an increase in BRI and its promise of greater cooperation significance. A consequence of this is to make BRI Health Cooperation standout in positive ways to policymakers, industry practitioners and patients. At the same time, most countries along B&R are yet to develop their economics, and the political and security situations in some of them continue to be turbulent [13]. Undoubtedly, these situations pose significant barriers for the development of local health services, one that outside sources can potentially remedy. In other words, the conditions are present in which a sustainable uptake of BRI Health Cooperation can be achieved, which is a point-of-view most notably cast by the “needs” of developing countries and “advantages” for a new player, such as China, to emerge on the global health stage.

### Priority “needs” of BRI health cooperation

Our findings highlighted several key areas that the BRI Health Cooperation should prioritize, which include health industry, medical and health services and infectious disease prevention and control. Unsurprisingly, these “needs” align with the “Healthy China 2030 Planning Outline” [14] and “Implementation Plan” [4]. Other important “need” areas concern health-related industries, specifically the strength of industry development and cooperation to produce and maintain, among many products, pharmaceuticals and medical devices, health foods, medical tourism and health information construction [15].

This medical and health services development and cooperation will likely stimulate consumer demand and promote the growth of service trade along B&R, which is undoubtedly a good way to engage populations while safeguarding their health [16]. An example of this cooperation is the hospital alliance between China and Central and Eastern European countries, where joint research and treatment of intractable diseases are engaged, allowing the respective publics to enjoy the highest level of medical diagnosis and treatment at home [17].

Other areas of development and potential cooperation include Chinese traditional medicine and protection and control of infectious diseases. Chinese traditional medicine technology and services are likely to evolve in relation to BRI Health Cooperation development, with Chinese medicine acupuncture already adopted and adapted in 183 countries and regions worldwide [18]. In terms of infectious diseases, globalization trends mean these diseases need to be taken more seriously; or the alternative is to risk a global health crisis. In the past decade, China has carried out a series of cooperation projects with Laos, Vietnam, Myanmar, and other countries along B&R that targeted malaria, dengue fever, AIDS, and other infectious diseases. Greater BRI Health Cooperation will inevitably expand these prevention and control projects, leading to stronger, more consistent health outcomes.

### Important factors in promoting BRI health cooperation

Significantly, the “needs” correspond to many of our findings for “advantages”. A summary of the key “advantages” highlights long-term partnerships, and good investment and financing systems. These advantages reflect the broader importance of taking a multi-layer approach to international cooperation. An illustration of how this is currently being achieved is the Asian Infrastructure Investment Bank (AIIB), which, since 2014, has become an embodiment of China’s international perspective of openness and economic reform, and, importantly, presents clearer institutional pathways for health to connect with diplomacy, economics, politics, and other fields of international exchange. This notion of layering is also useful when considering the function of already established cooperative partnerships. These partnerships will serve as a favorable model for other future cooperation activities [19]. Here, the idea for international cooperation is to prioritize partners with existing cooperation bases and ensure continued, smooth financial support.

In this context the acceptance of China as a key player as well as the relative locations of partner countries to China are significant issues to consider. Western governments and institutions may play a role in promoting BRI Health Cooperation, although the geographical distance from China means the specifics of their role is less clear. In contrast to this point, our findings showed that countries had low perceptions of geographical advantages and preferential policies. Even though the neighboring Asian countries are the starting countries of B&R, cross-border cooperation may remain a major challenge. Surprisingly, our results show that the Asian countries consider their geographical advantage to be weak, while both the African and Western countries consider it even weaker. These results are similarly reflected in terms of preferential policies. That is, despite China’s governments of all levels on international health cooperation issuing a series of relevant preferential policies, international awareness of these policies is still low (which may be related to the insufficient spillover effect of domestic preferential policies). It is advised that the Chinese government departments take the geopolitical gaps into account when developing a specific policy, publicize the policy to international partners based on sufficient understanding of cultural differences, so as to further facilitate deeper health cooperation among China and its partners.

### Major barriers of BRI health cooperation

It is found in our findings that both Asia and Western countries considered “Cultural differences” to be the biggest barrier for health cooperation. This barrier has been explored by some Chinese studies [20, 21]. In Africa, the biggest barrier was identified as “Communication platform”. More specifically, sector-oriented barriers were also analyzed, although similar results were found. For example, in the professional interaction process of diagnosis and treatment, culture is considered to be more significant than professional knowledge. In international trade activities, cultural differences are considered to be a factor in negative trade flow outcomes [22]. These results suggest that in international health exchanges, cultural differences in different countries may become a major barrier for cooperation. Undoubtedly, the lack of communication platforms, as highlighted in the African results of the survey, is a contributing factor in this barrier, particularly when considering the benefits of a continuous flow of information between international parties. In the same way, the main barriers identified by Europe and the United States, that is, a lack of international electronic trading platforms, define other barriers relating to “exchange”, albeit ones to do with trade instead of communication.

The differences in these barriers may derive from the differences in economic development of those areas [23]. In terms of resolving communication and e-commerce platform barriers, China’s participation in South-South cooperation and China-Africa cooperation provides a platform for international exchanges and cooperation, but the areas are limited to infrastructure construction, energy and environment, business development, human resources, health education, and so on [24]. Given this gap in platform coverage, it is clear a normalization mechanism for large-scale exchange activities in the health field should be established. It is also advisable that China make an effort to develop cross-border medical e-commerce platforms with countries in Europe and North and South Americas. It is conceivable that the Alibaba Group, for example, which established the mobile third-party payment tool “Alipay” when developing the Taobao system in 2003 [25], could be expanded to facilitate health-based trade. However, as noted above, it would mean an integrative process based on mutual understanding, including cultural and communicative factors. This returns the discussion to the key argument of this article: understanding the various factors, including “needs”, “advantages” and “barriers”, in international health cooperation is crucial.

### Limitations

The main limitation of the study was the sample size. The international participants in the conference were Ministers and higher rank officials. They provided a strong understanding of their national conditions and health development plans. However, the relatively small sample size meant it was difficult to avoid instances of personal opinion that could be deemed “biased”. Research that documents a larger sample size would help reduce the impact of this issue on the study. In addition, a greater number of countries would benefit the study, particularly when considering that BRI is an open concept involving all countries in the world.

## Conclusions

In this study, we have identified priority fields that China can collaborate with BRI Countries and show its leadership, such as health industry, medical services, and infectious disease prevention and control. It is found that successful health cooperation between China and other BRI countries is largely dependent on maximizing present health-industry partnerships, investment and financing, as well as overcoming cultural and communication barriers. These findings are all based on a developed understanding of China and its BRI Health Cooperation partners, their needs, and thoughts on cooperation advantages and barriers.

## Data Availability

The datasets used and/or analyzed during the current study are available from the corresponding author on reasonable request.

## Abbreviations

B&R: Belt and Road
BRI: Belt and Road Initiative
CDC: Centers for Disease Control
CMT: China Medical Team
